# Less is more: CRISPR/Cas9-based mutations in *DND1* gene enhance tomato resistance to powdery mildew with low fitness costs

**DOI:** 10.1186/s12870-024-05428-3

**Published:** 2024-08-10

**Authors:** Ruiling Li, Lei Cui, Matteo Martina, Valentina Bracuto, Fien Meijer-Dekens, Anne-Marie A. Wolters, Andrea Moglia, Yuling Bai, Alberto Acquadro

**Affiliations:** 1 Plant Genetics and Breeding, Department of Agricultural, Forest and Food Science (DISAFA), University of Torino, Grugliasco, 10095 Italy; 2 Plant Breeding, Wageningen University & Research, Wageningen, 6708 PB The Netherlands; 3 College of Agriculture, Shanxi Agricultural University, Taiyuan, 030031 China

**Keywords:** CRISPR/Cas9, S-genes, *dnd1* mutants, Reduced symptoms, Reduced fitness costs

## Abstract

Powdery mildew (PM), triggered by *Oidium neolycopersici*, represents a significant threat and a major concern for the productivity of tomato plants (*Solanum lycopersicum* L.). The presence of susceptibility (S) genes in plants facilitates pathogen proliferation and their dysfunction can lead to a recessively inherited broad-spectrum and durable type of resistance. Past studies have demonstrated that disrupting the function of *DND1 (Defense No Death 1)* increases plant resilience against various pathogens, such as powdery mildew (PM), but this comes at the cost of negatively affecting the overall health and vigor of the plant. To investigate the possibility of minimizing the adverse effects of the *dnd*1 mutation while boosting disease resistance, a CRISPR-Cas9 construct with four single guide RNAs targeting three exons of *SlDND1* (Solyc02g088560.4.1) was designed and introduced into the tomato variety Moneymaker (MM) through *Agrobacterium tumefaciens*-mediated transformation. Three T_1_ lines (named E1, E3 and E4) were crossed with MM and then selfed to produce T_F2_ families. All the T_F2_ plants in homozygous state *dnd1/dnd1*, showed reduced PM symptoms compared to the heterozygous (*DND1/dnd1*) and wild type (*DND1/DND1)* ones. Two full knock-out (KO) mutant events (E1 and E4) encoding truncated DND1 proteins, exhibited clear dwarfness and auto-necrosis phenotypes, while mutant event E3 harbouring deletions of 3 amino acids, showed normal growth in height with less auto-necrotic spots. Analysis of the 3D structures of both the reference and the mutant proteins revealed significant conformational alterations in the protein derived from E3, potentially impacting its function. A *dnd1/dnd1* TF2 line (TV181848-9, E3) underwent whole-genome sequencing using Illumina technology, which confirmed the absence of off-target mutations in selected genomic areas. Additionally, no traces of the *Cas9* gene were detected, indicating its elimination through segregation. Our findings confirm the role of *DND1* as an S-gene in tomato because impairment of this gene leads to a notable reduction in susceptibility to *O. neolycopersici*. Moreover, we provide, for the first time, a *dnd1* mutant allele (E3) that exhibits fitness advantages in comparison with previously reported *dnd1* mutant alleles, indicating a possible way to breed with *dnd1* mutants.

**Supplementary Information:**

The online version contains supplementary material available at 10.1186/s12870-024-05428-3.

## Introduction

Tomato (*Solanum lycopersicum* L.), whose berries are rich in lycopene, vitamins and minerals, is the world’s second most cultivated and profitable vegetable after potato. In the year 2020, over 186.8 million tons of tomatoes were harvested [1]. Tomato yields are extremely variable in the world, as the crop is challenged by abiotic stresses, like salt [2–4] and heat [5]; and biotic stresses, especially numerous diseases caused by fungi, bacteria, phytoplasmas, viruses and viroids [6]. Powdery mildew (PM), caused by ascomycete fungi belonging to the Erysiphales order, infects the aerial regions of higher plants and may reduce yield by up to 30% [7–9]. Among them, *Oidium neolycopersici* strongly infects tomatoes and causes powdery white lesions on the adaxial and abaxial leaf surfaces, petioles, and calyx. Severe infections lead to leaf chlorosis, premature senescence and considerable fruit size reduction and quality [10], posing a significant threat to tomato production [11]. As a result, much research effort has been focused on testing wild tomato species for their resistance to *O. neolycopersici* infection, which can be crossed with the commercial tomato to breed resistant new cultivars [12, 13].

To date, five dominant monogenic resistance loci, known as *Ol*-genes, have been discovered in wild tomato species. *Ol-1* (derived from *S. habrochaites* G1.1560), *Ol-3* (from *S. habrochaites* G1.1290), and *Ol-5* (from *S. habrochaites* PI247087) offer partial resistance through a delayed hypersensitive response (HR). Conversely, *Ol-4* (sourced from *S. peruvianum* LA2172) and *Ol-6* (of unknown origin) induce a rapid HR, resulting in complete resistance to powdery mildew (PM), which is race-specific [14]. Beyond these dominantly inherited *Ol*-genes, a recessive gene, *ol-2*, found in *S. lycopersicum* var. *cerasiforme* LA1230, provides broad-spectrum resistance to various PM species [15] when present in homozygous state. The cloning of *ol-2* showed it to be a homologue of the barley susceptibility (S) gene to powdery mildew, *MILDEW RESISTANCE LOCUS O (MLO)* [15]. In tomatoes, at least three *SlMLO* genes, *SlMLO1*, *SlMLO5*, and *SlMLO8* [16], are implicated in susceptibility to PM caused by *O. neolycopersici*. Of the three *MLO* homologues, the *SlMLO1* gene plays a major role since natural-occurring (the *ol-2* allele), EMS-induced (the *m200* allele) and CRISPR-induced loss-of-function mutants, are able to arrest almost completely fungal penetration and sporulation [15–18].

A plant gene facilitating a compatible interaction with a pathogen is termed an S-gene. These genes are part of a wide range of gene families, participating in various functions, many of which are crucial for plant physiological processes [19, 20]. Although mutations in S-genes can confer durable, recessive, and potentially wide-spectrum resistance to plants, their inactivation may result in pleiotropic effects [21, 22]. Apart from *ol-2* (impaired *MLO1* gene), there have been several instances where resistance to the PM fungus *O. neolycopersici* was attained through the dysfunction of S-genes in both tomato and *Arabidopsis*. An example includes *PMR4* (*Powdery Mildew Resistant 4*), which encodes a callose synthase responsible for callose production under (a)biotic stress conditions [23]. The use of CRISPR/Cas9-based gene editing has been successful in reducing susceptibility to late blight caused by *Phytophthora infestans* and to PM in tomato [24, 25]. *DMR1* (*Downy Mildew Resistance 1*) encodes a homoserine kinase (HSK). RNAi silencing of *DMR1* homologs in tomato reduced proliferation of *O. neolycopersici*, although *DMR1* silencing also caused dwarfing [26]. *DMR6* (*Downy Mildew Resistance 6*) encodes a 2OG and Fe(II)-dependent oxygenase with salicylic acid (SA) 5-hydroxylase activity, reducing the active SA pool. CRISPR mutants with inactivated *DMR6* in tomato show increased SA levels and enhanced resistance to different classes of pathogens, including *O. neolycopersici* [27–29]. *CESA3* (*Cellulose synthase 3*) encodes a subunit of the cellulose synthase complex which is essential for plant cell wall formation [30]. The *Arabidopsis cesa3* (*cev1*) mutant shows constitutively activated jasmonate (JA) and ethylene (ET) defence signalling pathways and has increased resistance to several species of PM, among which *O. neolycopersici* [31].

*Arabidopsis Defense No Death 1* (*AtDND1*) encodes a cyclic nucleotide-gated cation channel (CNGC; also known as AtCNGC2) [32–35]. The *Arabidopsis dnd1* mutant shows a reduced ability to produce an HR cell death response, a central feature of gene-for-gene plant disease resistance, while exhibiting enhanced resistance against a broad spectrum of fungal, bacterial, and viral pathogens [32, 33]. In previous studies, it was reported that RNAi-silencing of both tomato and potato orthologs of *DND1* resulted in resistance to *P. infestans* and *Botrytis cinerea* [36, 37] as well as to two PM species, *O. neolycopersici* (*On*) and *Golovinomyces orontii* [36]. However, the pleiotropic effects of *DND1* silencing have been underlined especially in tomato, showing a severe dwarf phenotype, auto-necrosis and decreased male fertility [36, 38]. Comparing the fitness costs between tomato and potato, it was found that potato *dnd1* RNAi-mediated knockdown (KD) mutants displayed a significantly weaker *dnd1* phenotype with much less dwarfism and fewer auto-necrotic spots, which may be due to the tetraploidy of potato [38]. To make *DND1* a highly reliable and valuable target for tomato breeding, it is thus crucial to propose a strategy to reduce fitness costs. CRISPR/Cas9 editing has emerged as a revolutionary tool in crop breeding, and has greatly facilitated the functional characterization of tomato genes involved in fruit yield and quality, stress response, development and ripening, and domestication process [13, 39, 40].

In this study, we report the successful generation of three different mutant alleles of the tomato ortholog of the *DND1* gene via CRISPR/Cas9 system and investigate the impacts of different mutation events on PM resistance and the fitness trade-off.

## Materials and methods

### Experimental scheme

**Fig. 1:**
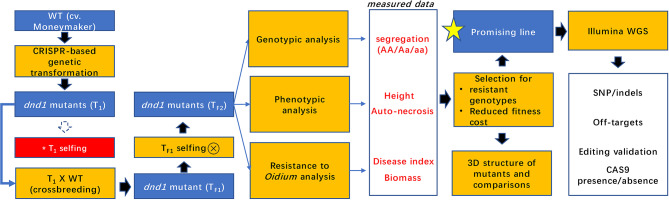
The experimental scheme used in this work for the identification of desirable *SlDND1* mutants that exhibited increased disease resistance and the lowest fitness cost. *T1 selfing did not yield any seeds

An experimental scheme (Fig. 1) was employed to generate stable mutants in the *DND1* gene with a reduced fitness cost. First, genetic transformation of the tomato cultivar ‘Moneymaker’ (MM) was conducted to introduce targeted mutations in the *dnd1* gene through CRISPR-based technology. T_1_ transformants were tested for powdery mildew response and genotyped with a NPTII marker. Several T_1_ transformants showing less powdery mildew infection were selected and crossed with MM (T_1_ x MM), to produce a T_F1_ generation. T_F1_ plants were selfed to produce a T_F2_ generation. T_F2_ plants were phenotyped, genotyped, and analysed for disease resistance. All T_F2_ families used in this study are shown in Table 1.

**Table 1:** CRISPR-induced mutation events with their phenotypes and mutations. Columns B-E refer to PCR fragments as indicated in Fig. 2. Fragment B includes sgRNA10, fragment D includes sgRNA5 and sgRNA6 and fragment F includes sgRNA8; mutations at target sites indicated as + n bp (insertion) or -n bp (deletion); *represents a frameshift mutation resulting in a truncated protein. The full length SlDND1 protein (annotation Heinz ITAG4.1) contains 708 amino acids

Event	Phenotype	length (aa)	B(sg10)	C	D(sg5)	D(sg6)	E	F(sg8)
**E1**	severe dwarf, severe necrosis	381	-	-	-3 bp	+ 1 bp*	-	-
**E3**	no dwarf, necrotic spotting at edge of leaves	705	-3 bp	-	-	-6 bp	-	-
**E4**	severe dwarf, severe necrosis	129	-2 bp*	-	-	-1 bp/-8 bp/none	-	− 7 bp

### CRISPR/Cas-9-targeted mutagenesis of *SlDND1*

Four single guide RNAs (sgRNAs) (sgRNA10: GAAGCAAGCGCGTGCAGAGA, sgRNA5: ATGTGTTTGGATGTCAATGG, sgRNA6: GTCAATGGACCATTTCCATA, sgRNA8: GCCACAAGCATACTTGAGCC) were designed targeting the *DND1* homolog (Solyc02g088560.4.1) from the Sol Genomics Network database [41], on the website https://cctop.cos.uni-heidelberg.de/, and selected manually as described by [42, 43]. The program Cas-OFFinder (http://www.rgenome.net/cas-offinder/*)* was used to check for possible off-targets of the four sgRNAs of *SlDND1*. The mismatch number was set at 3 or less. A single CRISPR/Cas9 construct containing the four sgRNAs, the *NPTII* resistance gene, and the *Cas9* gene was constructed as described by [25]. The plasmids were cloned using *E. coli* DH5α and transformed to *Agrobacterium* strain AGL1. MM was used for genetic transformation according to the method described by [44]. Primary transformants (T_1_) were obtained from the in *vitro* cultivation, and the mutants positive for both *NPTII* and *Cas9* gene were selected. Since it was difficult to obtain selfing seeds from the *dnd1* T_1_ mutants, a T_F1_ generation was produced by crossing T_1_ and wild-type MM plants.

### PCR-based characterization of mutation events and genotyping

**Fig. 2:**
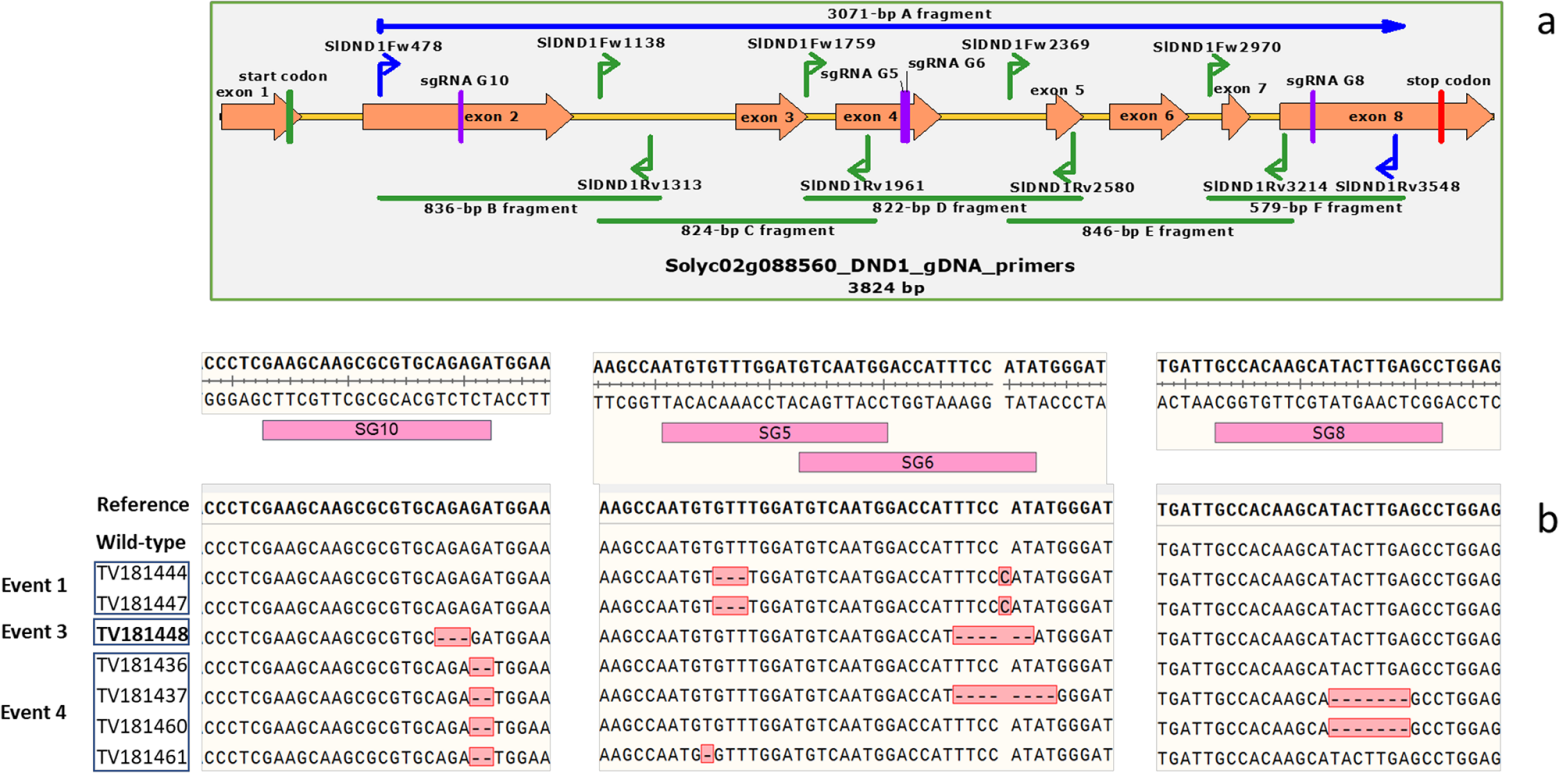
Position of target sites of the sgRNAs in *SlDND1* (Solyc02g088560.4.1) and editing details of CRISPR events. (**a**) Graphical representation showing the locations of the sgRNAs’ target sites in pink, primers flanking all target sites in blue, and primers for amplification of smaller overlapping regions in green. The 3071-bp region of *SlDND1* (fragment A) containing 4 sgRNAs was divided into fragments named B, C, D, E, and F for Sanger sequencing and identification of the mutations. (**b**) Mutations in T_F2_ families from 3 different editing events. The results were obtained from Sanger sequencing

DNA was extracted from the T_1_, T_F1_ and T_F2_ genotypes with the modified CTAB DNA extraction method (Porebski et al. 1997), quantified on the Qubit fluorometer (Thermofisher, USA), and NanoDrop™ One Microvolume UV-Vis Spectrophotometer (Thermofisher, USA). We used 5 pairs of primers covering all the regions of *SlDND1* with possible mutations that could be visible via electrophoresis. The positions of these primers and their PCR products are shown in Fig. 2, and primer sequences are provided in Table S1. The distinction between homozygous mutant, heterozygous, and homozygous wild-type T_F2_ plants was made by PCR using the same primers as described above. The amplified fragments were Sanger sequenced, and their allelic status (monoallelic, bi-allelic, and heterozygous) was determined by TIDE (Tracking of Indels by DEcomposition) at http://shinyapps.datacurators.nl/tide.

### *Sldnd1* mutant phenotype analysis and disease assay

Seeds from both T_F1_ and T_F2_ families were sown individually, with the resulting plant numbers for each family detailed in Table S2. The greenhouse conditions at Unifarm of Wageningen University & Research (The Netherlands), were maintained at 21 ℃ during the day and 19 ℃ at night, alongside a relative humidity of 70% ± 15% and a photoperiod of 16 h. At the age of four weeks, the plants were treated with a spray of *O. neolycopersici* (*On*) conidiospores, sourced from the leaves of infected tomato MM plants, at a density of 3.5*10^4^ spores per milliliter. Disease index (DI) evaluations were conducted 10 and 12 days post-inoculation by visually assessing the powdery mildew symptoms on a scale ranging from 0 to 3 [15]. For comparison, wild-type MM plants served as the control group (Ctrl).

### Quantification of relative fungal biomass

At 21 days after inoculation, the fourth true leaf was harvested from each of the infected tomato mutants and the control plants. The genomic DNA (gDNA) of both the plant and the fungus was extracted from these samples using a modified CTAB method [45]. The primers aimed at quantifying fungal biomass, as listed in Table S1, were designed to target the internal transcribed spacer (ITS) region of *O. neolycopersici* (*On*) and *the Elongation Factor 1α (Ef1α)* gene of the tomato [16, 46]. Quantitative real-time PCR (qRT-PCR) analyses were conducted on three biological replicates using a C1000 light cycler system (Bio-Rad) and SYBR Green mix (Bio-Rad). The relative quantity of fungal to tomato gDNA was determined using the 2^−ΔΔCt^ method [47].

### Whole genome sequencing of wild-type MM and TV181448 mutant

A genomic DNA library with short inserts (350-bp) was constructed using one microgram of DNA (Novogene, Hong Kong) and sequenced on an Illumina sequencer (Illumina Inc., San Diego, CA, USA) employing paired-end sequencing technology (2 × 150 bp). The initial sequencing data were processed using fastp (https://github.com/OpenGene/fastp) to eliminate any remaining adapter sequences and low-quality reads (Q < 30). Following this, a de novo assembly of the genome was carried out with the MegaHit assembler (version 1.2.9, https://github.com/voutcn/megahit), applying specific parameters for the assembly process (k-min = 27, k-max = 141, k-step = 10, cleaning-rounds = 1, and disconnect-ratio = 0). To evaluate the assembly quality, metrics such as N50, the number and size of contigs/scaffolds, and the total genome length were calculated using the Assemblathon_stats.pl Perl script (https://github.com/ucdavis-bioinformatics/assemblathon2-analysis) (Table S3). Further analysis of the mutant’s genome sequence was performed using BLAST to search for potential insertions with the T-DNA sequence as the reference. The analysis prioritized results based on the e-value (e-value < 1 × e^− 10^), percentage of similarity, and coverage of the query.

### Homology modelling of DND1 (wild type and mutants) and comparison of 3D structures

The DND1 proteins in both wild-type (WT) and mutant forms were generated using the ‘getorf’ utility (http://emboss.sourceforge.net), and their sequences were aligned using Clustal Omega (https://www.ebi.ac.uk/Tools/msa/clustalo). The potential impact of mutations on the proteins’ functionality was assessed using the Provean server (http://provean.jcvi.org), which evaluates the functional effects of various types of protein sequence alterations, including single amino acid changes, insertions, deletions, and multiple substitutions, with a standard score threshold of − 2.5. For both WT and mutant proteins, homology models were created using SWISS-MODEL (https://swissmodel.expasy.org), based on the HCN1 channel structure from *Homo sapiens* L. (SMTL ID 6uqf.1) as the reference. These models underwent validation checks with Molprobity [48] and QMEAN [49]. The comparative analysis of the models was executed using UCSF Chimera (version 1.16, https://cgl.ucsf.edu/chimera).

### On- and off-target analyses and SNP statistics

In edited plants, the analysis of genomic variations and allele frequencies at the *SlDND1* locus was conducted using CRISPResso2 (http://crispresso2.pinellolab.org) for CRISPR edits and SNP/indel examination. Sequencing reads from the edited tomato plants were aligned to the tomato reference genome (SL4.0, https://solgenomics.net) utilizing the Burrows–Wheeler Aligner (version 0.7.17, https://sourceforge.net/projects/bio-bwa/files) with the ‘mem’ option and standard settings. The resulting BAM files were further processed for SNP identification using Samtools (version 1.9-166-g74718c2) mpileup, adhering to default settings but with a minimum mapping quality set to 20. This process generated a variant call format (vcf) file. The vcf file was then scrutinized within a 200 bp range of each sgRNA target site to identify SNP/indels, employing bedtools intersect (https://bedtools.readthedocs.io) for this purpose. For detecting potential off-target effects, the CasOT tool (https://github.com/audy/mirror-casot.pl) was employed to scan the tomato genome (SL4.0, https://solgenomics.net) for off-target sites, considering all sgRNAs as probes in sgRNA mode with the default PAM type (NGG = A) and allowing up to two mismatches in both the seed and non-seed regions. The identified off-target genomic regions were then cross-referenced with the vcf file using bedtools, for both edited and control plants, to exclude regions without polymorphisms in the controls. Custom bash scripts were utilized for the analysis of these results.

## Results

### CRISPR/Cas-9-targeted mutagenesis of *SlDND1* and mutant generation

A single CRISPR/Cas9 construct containing four sgRNAs (Fig. 2), the *NPTII* resistance gene, and the *Cas9* gene was built and used to transform the tomato cultivar MM, susceptible to *On*, via *A. tumefaciens*. The four sgRNAs were specific to the *DND1* homolog (Solyc02g088560) from the Sol Genomics Network database [41], increasing the possibility of obtaining large deletions between sgRNAs and disrupting the gene structure and function. Primary transformants (T_1_) were obtained following genetic transformation and in vitro cultivation, and a total of 39 T_1_ plants were selected via PCR screening for the presence of both *NPTII* and *Cas9* genes. Progenies that exhibited no PCR signal were tentatively considered to be *Cas9*-free. These transformants were tested with *On.* A subset of 12 transformants showing no or fewer powdery mildew symptoms was selected, of which all but one showed dwarfness and auto-necrosis.

### Phenotypes of the *Sldnd1* mutants

**Fig. 3:**
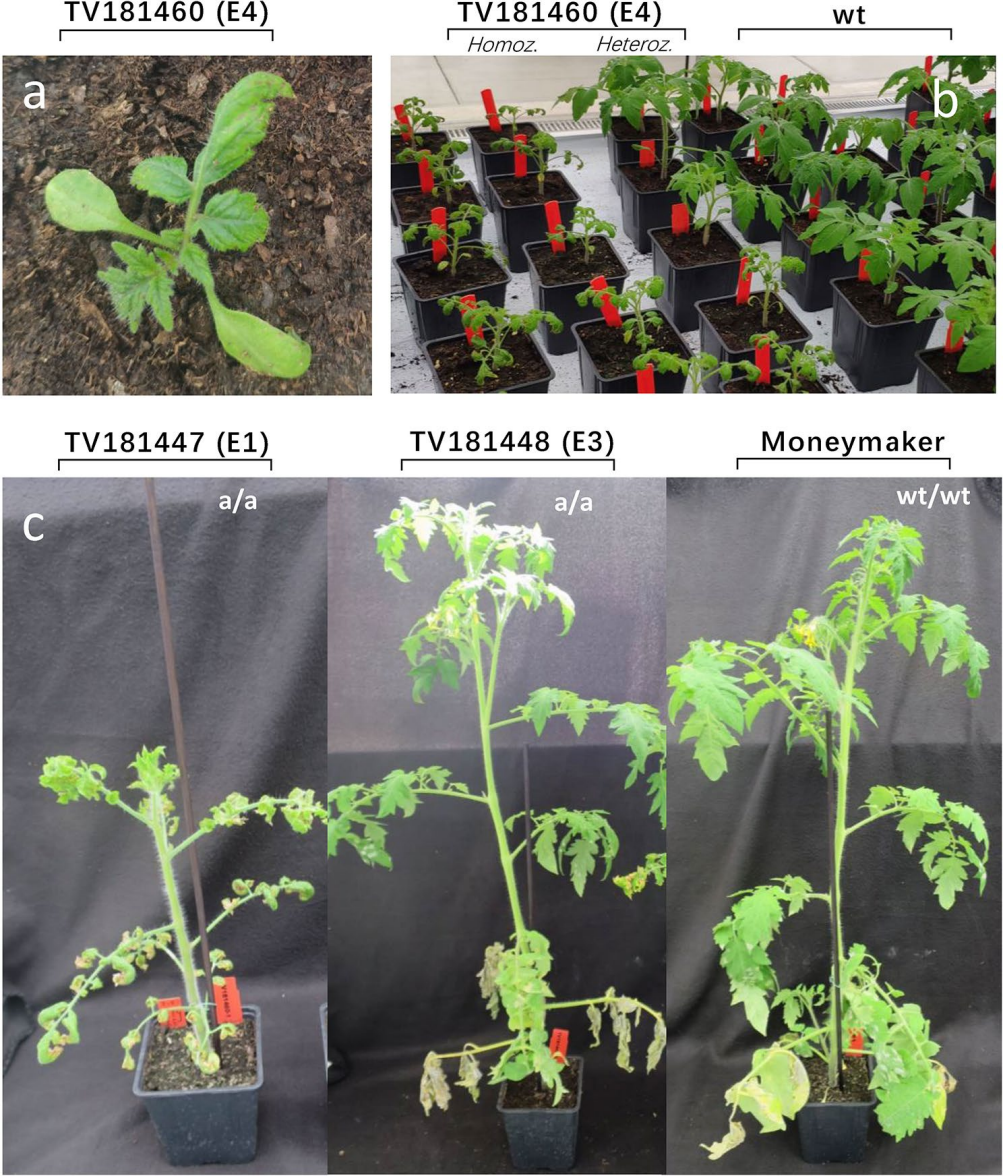
Phenotypes of some T_F2_*dnd1* mutants. (**a**) Auto-necrosis phenotype of young seedlings of the TV181460 T_F2_ family. Auto-necrotic spots were observed on the edge of its first pair of true leaves. The photo was taken 7 days after sowing. (**b**) Dwarf phenotype observed on young homozygous mutant TV181460 seedlings compared with heterozygous and wild-type plants. The photo was taken 15 days after sowing. T_F2_ family TV181460 was selected as representative of two KO mutation events, E1 and E4, with similar phenotypes. (**c**) Phenotypes of mature plants. Height comparison among the two *dnd1* homozygous T_F2_ plants and the wild-type one month after sowing. On the left a homozygous mutant of the TV181447 T_F2_ family is shown exhibiting a severely dwarfed phenotype; in the middle a homozygous mutant of the TV181448 T_F2_ family is shown, exhibiting a slight autonecrotic phenotype (E3); on the right a wild-type ‘Moneymaker’ plant is shown

Three specific T_1_ mutants (E1, E3, and E4) were selected as representatives based on their phenotypes: E1 and E4 exhibited clear dwarf and auto-necrosis phenotypes, while E3 showed a mild dwarf phenotype. These T_1_ plants were crossed with MM (Fig. 1). The obtained T_F1_ were selfed to generate T_F2_ progenies, segregating for the mutant *DND1* alleles. Among them, we observed distinct *dnd1* phenotypes (e.g.: plant height, Table S4). T_F2_ from E1 and E4 showed segregation of severely dwarfed plants with necrotic spots or normal plants (Fig. 3a, b), while plants derived from E3 were normal plants or exhibited a slightly dwarfed phenotype with fewer auto-necrotic spots, which appeared later than in E1 and E4 mutant plants (Fig. 3c).

### Genotypes of the *Sldnd1* mutants

The T_F2_ mutants were fully characterized through Sanger sequencing, and TIDE analyses allowed the reconstruction of the original editing events. Genotypes were amplified with primer pairs (Table S1) flanking the sgRNA target regions (five regions: B, C, D, E, F; Fig. 2). No large deletions were discovered at this step, compared with the length of the amplicons of wild-type plants. All T_F1_ plants were shown to be heterozygous (*DND1*/*dnd1*, Aa). The T_F2_ lines derived from the three events exhibited different allelic profiles (Table 1), segregating at the *DND1* locus (AA, Aa, and aa; Table S4). In particular, the mutant T_F2_ plants derived from the E1 event showed a 3-bp deletion at sgRNA5 and 1-bp insertion at sgRNA6, the latter generating a truncated protein. The mutant T_F2_ plants derived from the E3 event showed a 3-bp deletion at sgRNA10 and a 6-bp deletion at sgRNA6, resulting in the deletion of 3 amino acids. The mutant T_F2_ plants derived from the E4 event showed a 2-bp deletion at sgRNA10, generating a truncated protein, followed by a 1-bp or 8-bp deletion at sgRNA6 and a 7-bp deletion at sgRNA8. T_F2_ progenies underwent PCR screening to detect the presence or absence of both *NPTII* and *Cas9* genes (Table S1). Progenies that exhibited no PCR signal were tentatively considered to be *Cas9*-free.

### Resistance to powdery mildew in *Sldnd1* mutants

**Fig. 4:**
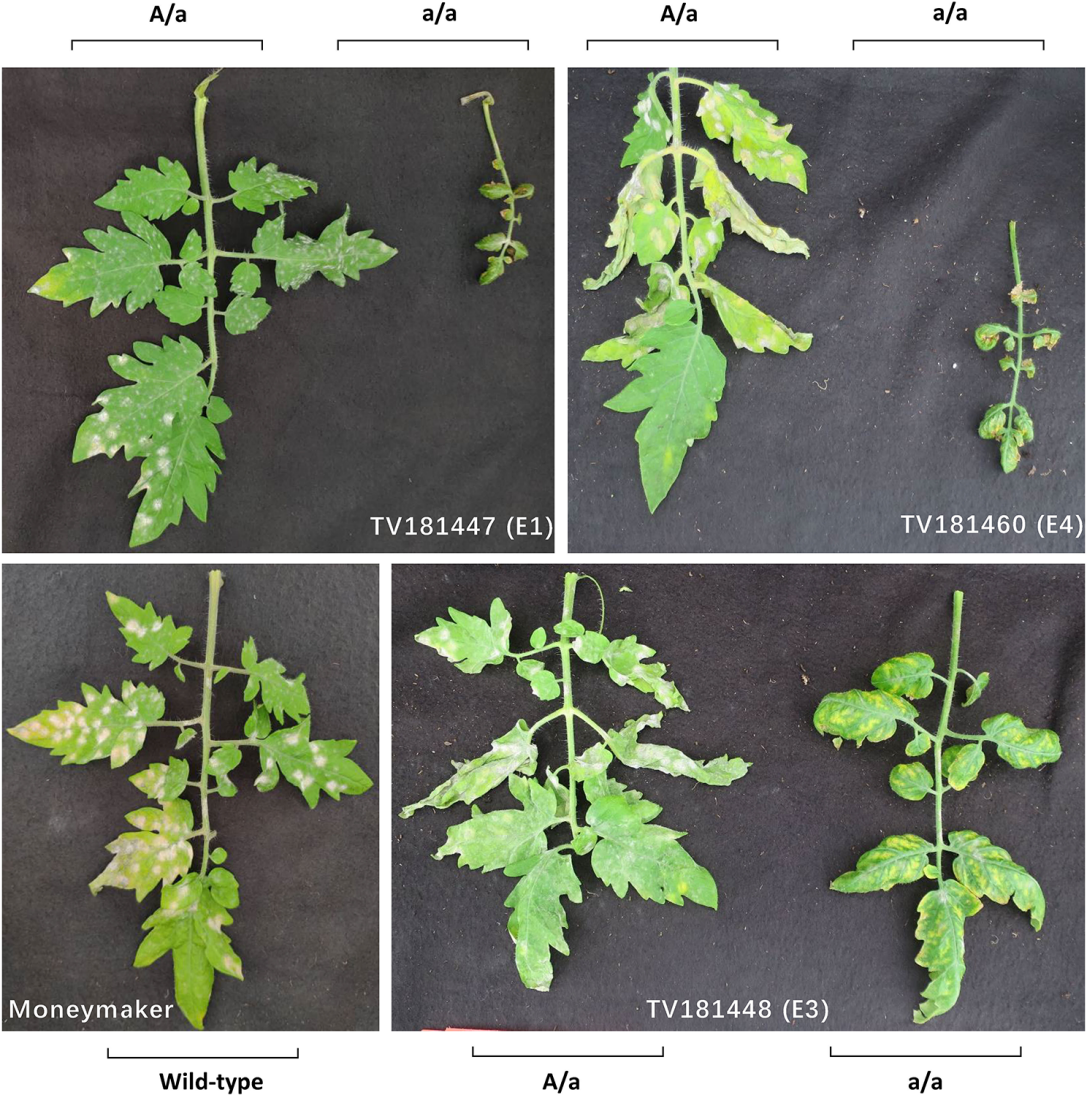
Phenotypic response to infection with *Oidium neolycopersici* of homozygous/heterozygous *dnd1* T_F2_ plants. Powdery mildew symptoms were observed on the leaves of both homozygous and heterozygous plants of each event (one T_F2_ family is given from each of the 3 mutation events; E1, E3, E4). Photos were taken 21 days post inoculation (dpi)

**Fig. 5:**
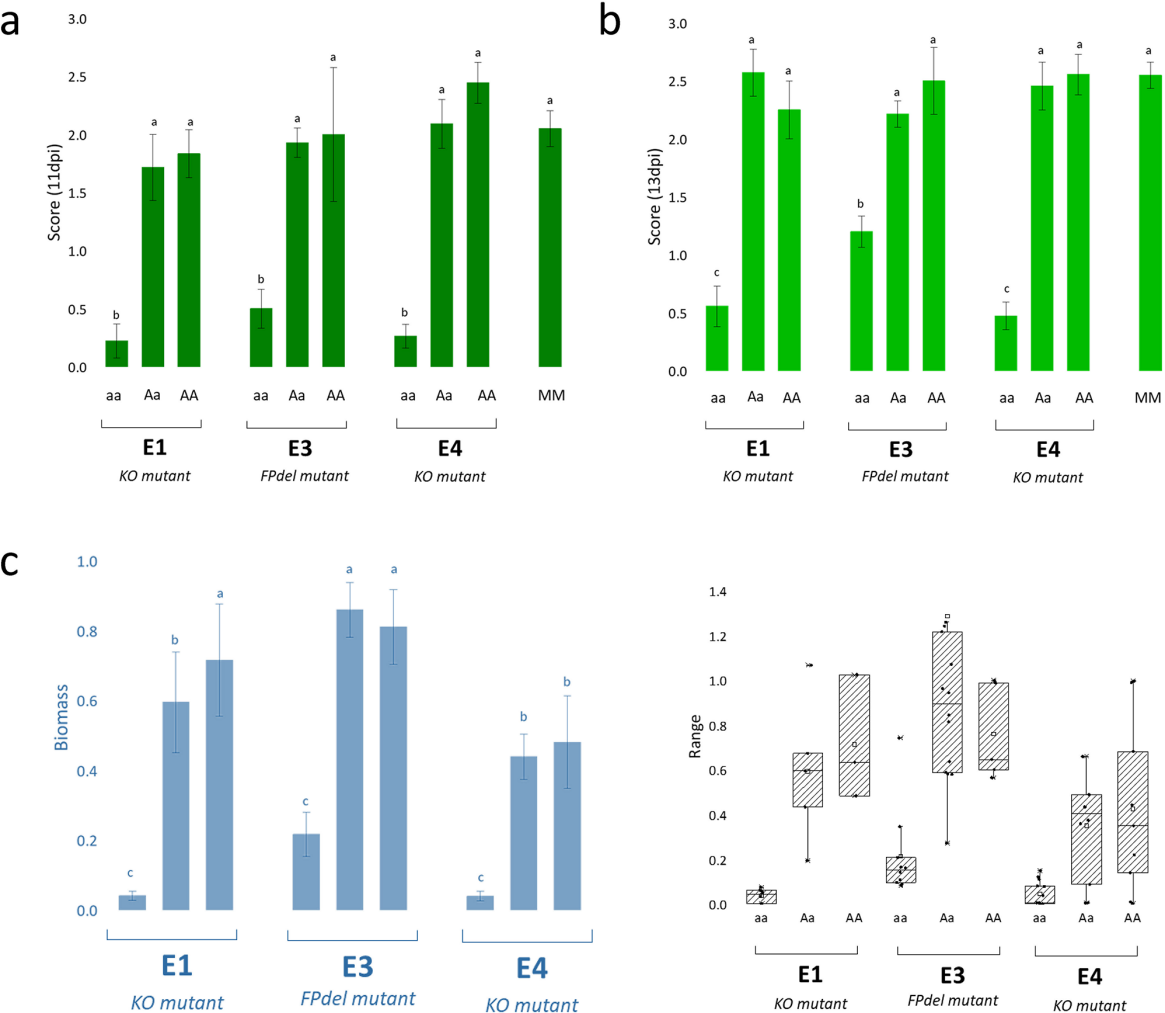
Average disease index score of the *dnd1* mutant plants in T_F2_ at 11 dpi (**a**) and 13 dpi (**b**). Homozygous mutants (aa) appeared significantly resistant in all the assayed events (E1, E3, E4); however, heterozygous mutants showed to be susceptible in all assayed events (E1, E3, E4). Moneymaker (MM) was used as control. c) Relative fungal biomass quantification on at least three individual plants of the mutant families (left, histogram; right, box plot diagram). Fungal biomass was calculated as the ratio of fungal *ITS* gene amplification in comparison with tomato *EF1a* and normalized with the values of the wild-type Moneymaker. Samples for the biomass were taken at 21 days post inoculation (dpi). The y-axis shows the mean ratio of the score of the mutant/control group; bars represent standard error. Statistical differences were analyzed with a two-tailed t-test (*p* < 0.05). KO mutant, knock-out mutant; FPdel mutant, mutant with deletion of amino acids F and P

To evaluate the resistance of the *Sldnd1* T_F2_ mutants, we inoculated them with *On* (Fig. 4), assessing the disease index (DI) score (Fig. 5a). Additionally, we quantified the disease severity by measuring the relative *On* biomass in the mutants, complementing the DI observations (Fig. 5b). MM plants were used as controls. Of the three T_F2_ families, all wild type (AA) and heterozygous (Aa) plants from E1, E3, and E4 were shown to be susceptible to *On*, with no significant differences in the DI score and the relative fungal biomass compared with the MM control (Fig. 5, Table S4). Homozygous mutants (aa) from E1, E3 and E4, however, were resistant to *On*, with a significant reduction in both DI score and the relative fungal biomass compared with Aa and AA plants as well as the MM control. Surprisingly, the aa mutants from E3, besides displaying improved resistance (Fig. 5), exhibited less dwarfism and auto-necrotic spots (Fig. 3c).

### Impact of *Sldnd1* copy number on plant fitness

**Fig. 6:**
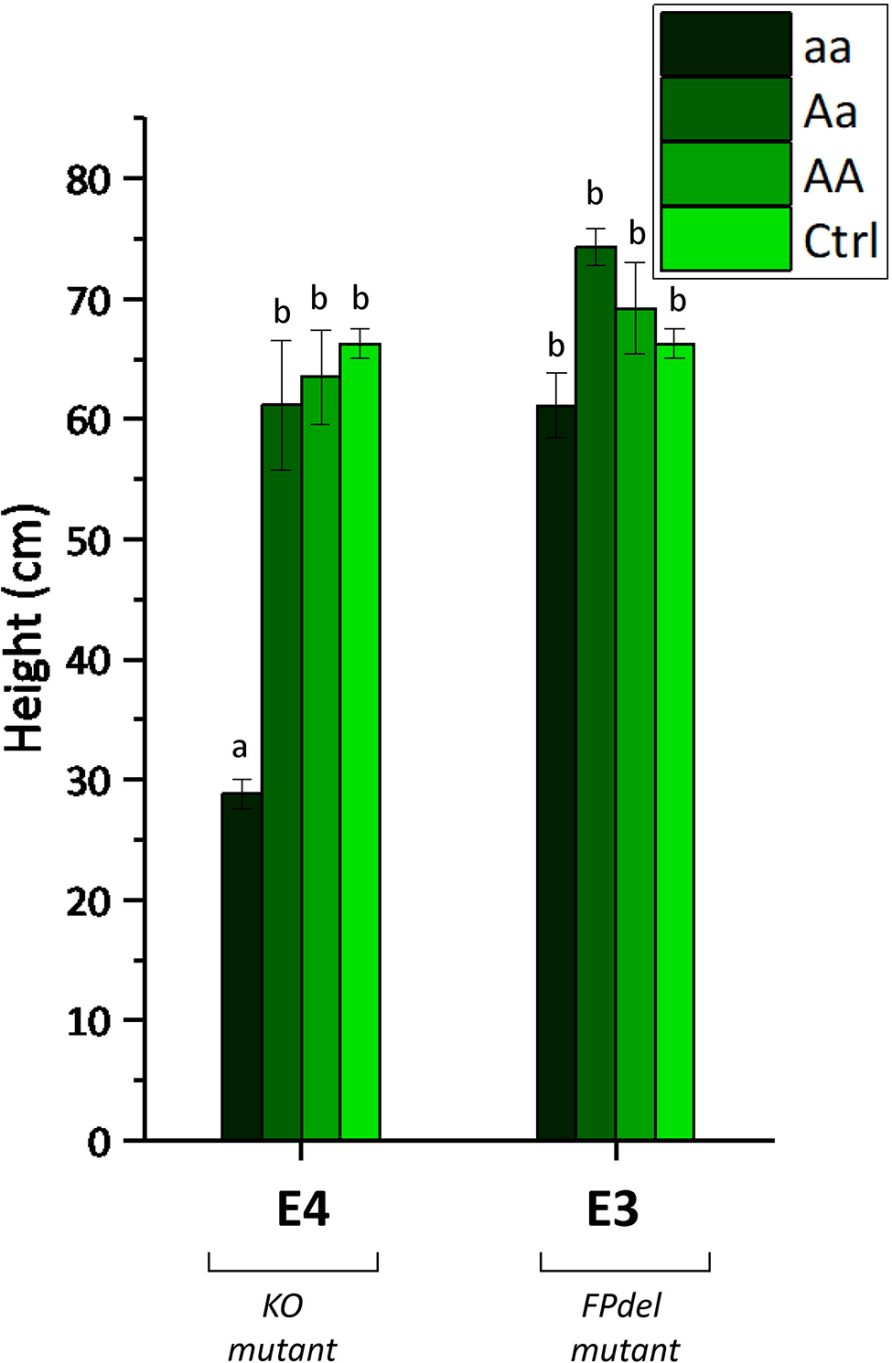
Height of homozygous/heterozygous *dnd1* and wild-type T_F2_ plants. Differences are shown between a dwarf event (E4), and the slightly dwarfed event (E3) in three different allelic states (aa, Aa, AA), compared with wild-type Moneymaker plants used as control (Ctrl), measured 7 weeks after sowing. KO mutant, knock-out mutant; FPdel mutant, mutant with deletion of amino acids F and P

To address the question of whether the *DND1* gene plays a dosage-dependent role in regulating PM resistance and fitness costs in tomato, we compared heterozygous plants of the segregating T_F2_ families with the homozygous plants. Heterozygous plants (one copy of the *dnd1* mutant allele) could combine a reduced fitness cost with an acceptable degree of disease resistance if *dnd1* functions in a dosage-dependent manner. For T_F2_ families derived from E1 and E4 at the seedling stage (till the 4th true leaf, 30 days after sowing), the *dnd1* (aa) homozygous plants displayed a significant reduction in size compared with the plants belonging to the other two genotype classes (Aa and AA). Interestingly, all heterozygous plants, including those from T_F1_ and T_F2_ generations, were intermediate in height and showed a moderately reduced *dnd1* phenotype (less dwarfism) without displaying auto-necrotic spots (Fig. 3b). At a later growth stage (two months after sowing; Fig. 6), the homozygous mutants (aa) from the E4 T_F2_ family showed statistically significant dwarfism (mean: 28.81 ± 1.23 cm), whereas the size of heterozygous plants (Aa) (mean: 61.15 ± 5.39 cm), was comparable with that of the plants carrying wild-type alleles (AA, mean: 61.20 ± 5.23 cm) and controls (MM, mean: 66.30 ± 1.28 cm). Notably, homozygous mutants (aa) from E3 showed a minor decrease in size (mean: 61.10 ± 2.69 cm), with no statistical differences when compared with heterozygous plants (74.33 ± 1.57 cm) and with those containing the wild-type alleles (AA; 69.25 ± 3.81 cm). The aa plants from E3 also showed fewer auto-necrotic spots, which appeared later than those of the *dnd1* homozygous mutants from the E1 and E4 families (Fig. 3c). A homozygous plant from the E3 T_F2_ family, designated as TV181448-9, was chosen for further genomic research.

### Whole genome sequencing of TV181448 (E3) and off-target effects

Illumina whole-genome sequencing was performed on TV181448-9 and a wild-type MM in vitro plant, producing 438 million raw paired-end reads (65.8 Gb; Table S3). The coverage varied between 43.4X for TV181448-9 and 40.6X for the wild type. Following the filtering and trimming process, we secured 436 million high-quality reads, accounting for 99.46% of the total. The sequence data were deposited in the NCBI Short Read Archive under specific submission identifiers (PRJNA1090062). A *de novo* genome assembly was carried out for the mutant T_F2_ plant (Table S3), and a blastn analysis revealed no T-DNA insertions in the scaffolds, indicating *Cas9* elimination by segregation. The mutation in the *DND1* locus (Solyc02g088560) of the T_F2_ plant TV181448-9 was confirmed, revealing a 3-bp deletion at sgRNA10 (Q114del) and a 6-bp deletion at sgRNA6 (FP369-370del), both in the homozygous state.

To verify that TV181448-9 displayed mutations solely in the *DND1* locus and to investigate potential off-target effects, we examined candidate off-target loci using the sequencing data. We identified a list of 28 potential off-targets for the four sgRNAs used to target the *DND1* locus, which all had more than 2 bp mismatches compared with the gRNAs, and were located in both coding (2) and non-coding (26) regions (Table S5 and S6). We mapped the Illumina reads from the wild-type and TV181448-9 genomes to the tomato ‘Heinz 1706’ reference genome for off-target analysis. All 28 putative off-target regions were fully covered by Illumina reads in both the wild type and *dnd1* mutant, ruling out the possibility of large deletions (data not shown). Comparing DNA alignments in the wild-type MM and *dnd1* mutant, we found no SNPs/indels in the candidate off-target regions. While some indels/SNPs were present in the surrounding regions (SL4.0ch05:26816411–26,816,434 and SL4.0ch12:31973239–31,973,262), they did not indicate off-target effects, being conserved SNP/indels between the *dnd1* mutant and wild-type MM or outside of the 20 bp window related to the gRNA-like sequence (200 bp window). These analyses confirm the specificity of Cas9-mediated *DND1* gene editing and demonstrate the absence of off-target effects. We detected 49,599 SNPs in TV181448-9, with 90.7% being heterozygous, and 43,757 SNPs in the wild-type Moneymaker (MM), with 89.3% heterozygosity, referencing the Heinz tomato genome. The mean number of SNPs and the rate of variation were similar between the edited and non-edited plants, exhibiting average variation rates of 6.34 × 10^− 5^ and 5.59 × 10^− 5^, respectively (Table S7).

### Homology modelling of DND1 in E3 event and 3D structure comparison

**Fig. 7:**
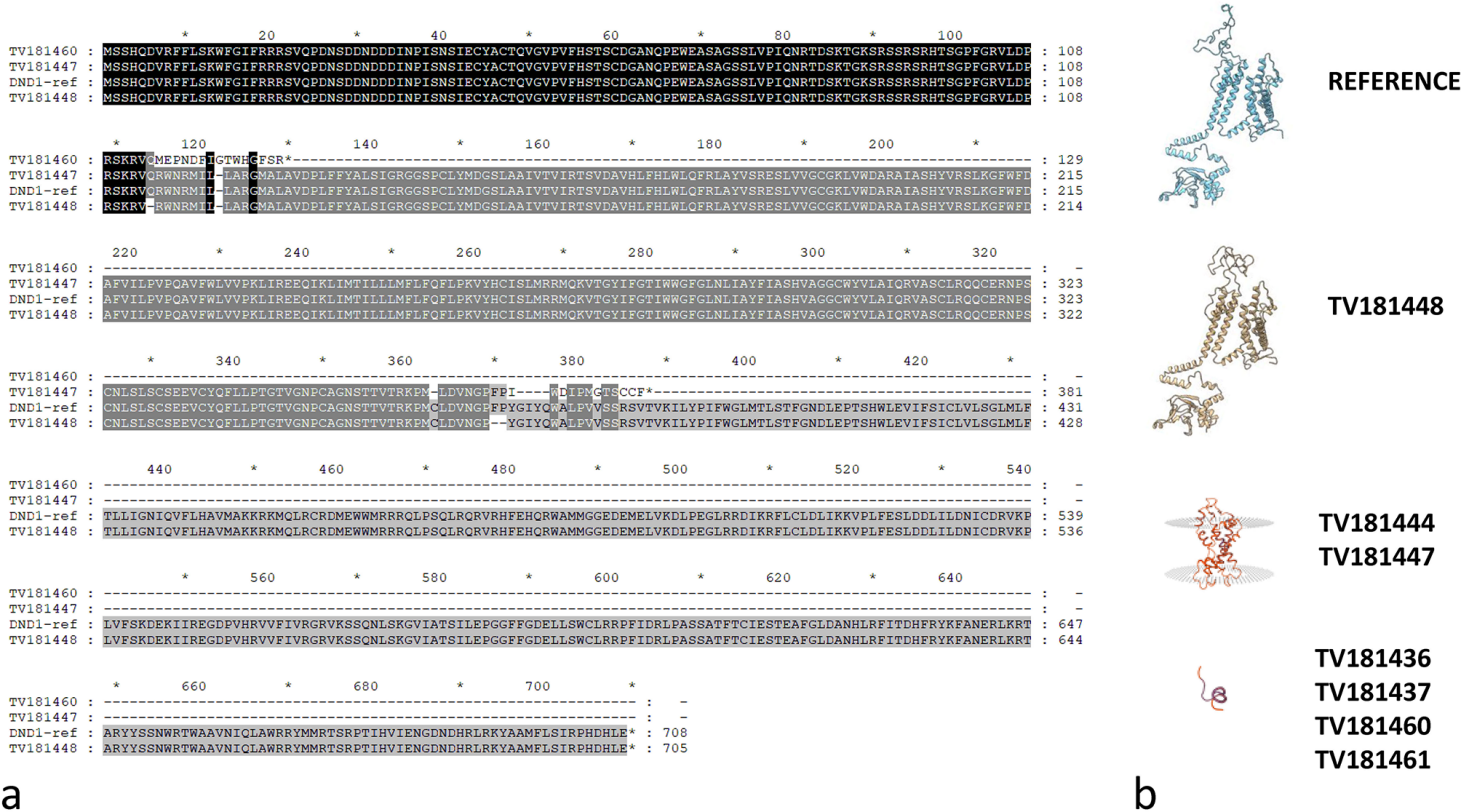
(**a**) Sequence alignment of the DND1 proteins using the ‘Heinz’ sequence as reference (DND1-ref) and predicted proteins for the different mutants; the protein sequences of ‘Heinz’ and ‘Moneymaker’ are identical, as determined from whole-genome sequencing (WGS) data comparison (data not shown). Dashes represent lacking amino acids in the mutants. (**b**) Predicted protein structures of wild-type and mutant DND1 proteins

**Fig. 8:**
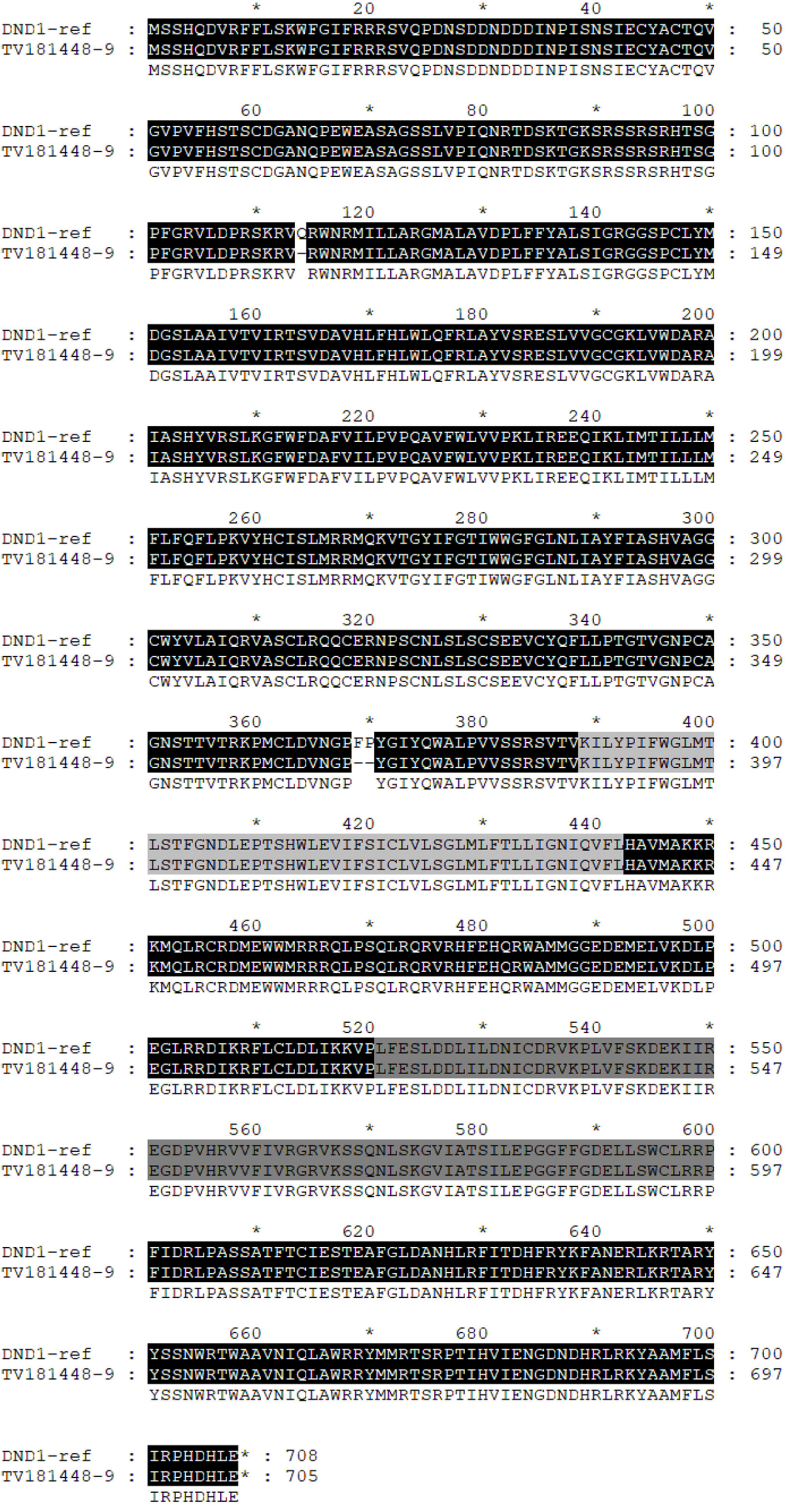
Sequence alignment of the DND1 proteins of ‘Heinz (SL4.0 reference, ITAG4.1) and *dnd1* mutant plant TV181448-9; the protein sequences of ‘Heinz’ and ‘Moneymaker’ are identical, as determined from whole-genome sequencing (WGS) data comparison (data not shown). Dashes represent deleted amino acids in the mutant. In light grey, “Selectively filter” and “Pore domain” are indicated; in dark grey, “Cyclic nucleotide Binding domain”

Preliminary sequence evaluation of the *dnd1* mutants was performed. Multiple sequence alignment (Fig. 7) of the reference protein (DND1, Solyc02g088560.4.1) with the predicted proteins of the 3 mutation events (Table 1) revealed two different types of protein changes: (1) severely truncated DND1 proteins (E1, E4) representing KO mutations, and (2) almost full-length protein without frame shift with 1 and 2 amino acids deleted (E3). Multiple sequence alignment (Fig. 8) of the E3 allele (in plant TV181448-9) with the reference protein (SlDND1, Solyc02g088560.4.1) revealed that the editing outcome (a 3-bp deletion at sgRNA10 and a 6-bp deletion at sgRNA6) resulted in amino acid deletions (Q114del and FP369-370del).

The mutation impact analysis on E3 protein functionality was conducted through the Provean server. The first nucleotide indel (-3 bp) was predicted to produce an amino acid deletion (Q114del), which Provean reported as highly deleterious (score − 8.657). The second nucleotide indel (-6 bp) was predicted to produce further amino acid deletions (F369_P370del), which Provean reported as highly deleterious (score − 9.486). Overall, the three amino acid deletions showed a high impact on protein function.

**Fig. 9:**
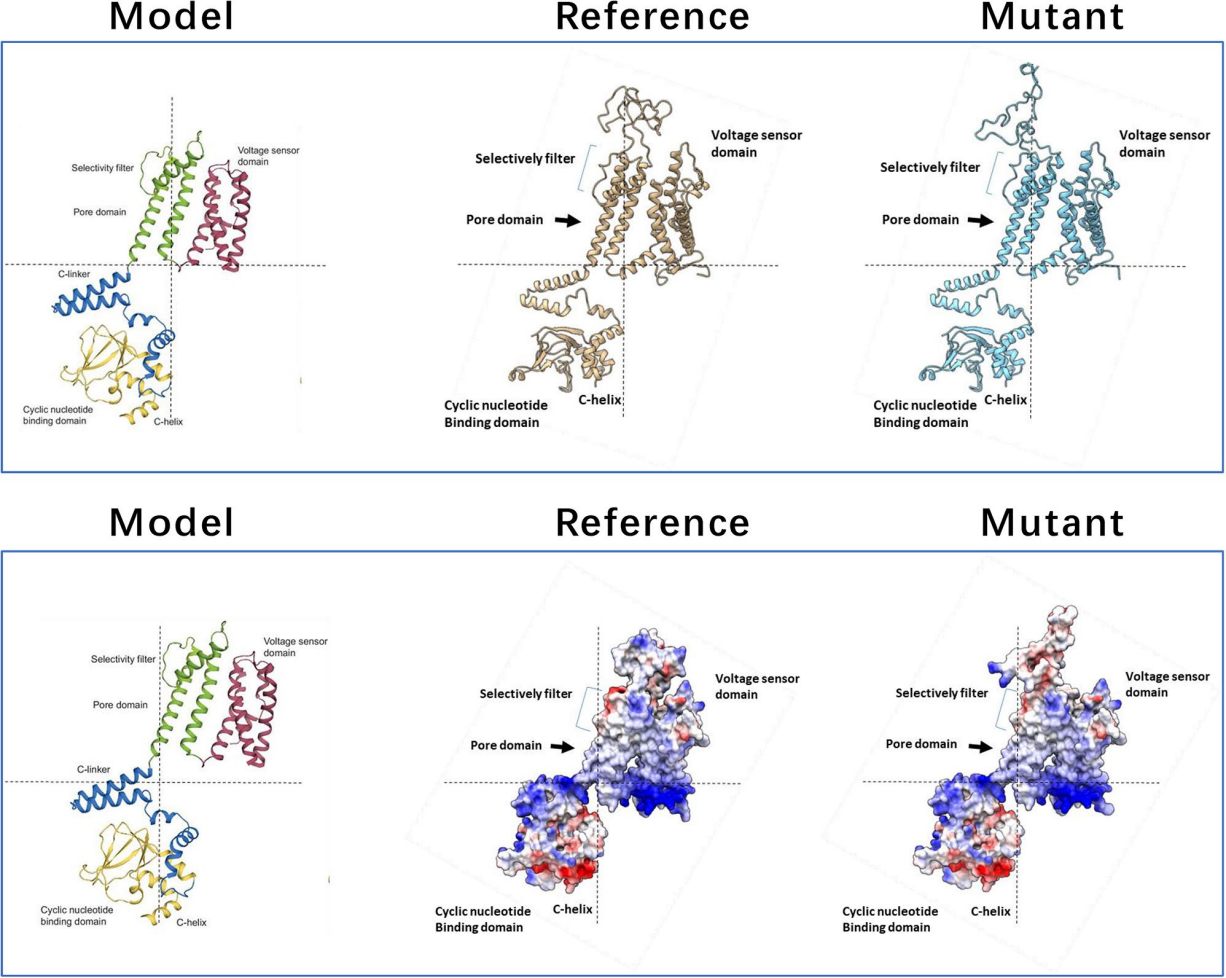
Comparison of 3D structures of DND1 (monomer) for the reference protein and the TV181448-9 mutant. Top: ribbon model; Bottom: electrostatic mode (red: positive charges; blue: negative charges; white: no charges). The annotated model protein is from Rheinberger et al. (2018)

**Fig. 10:**
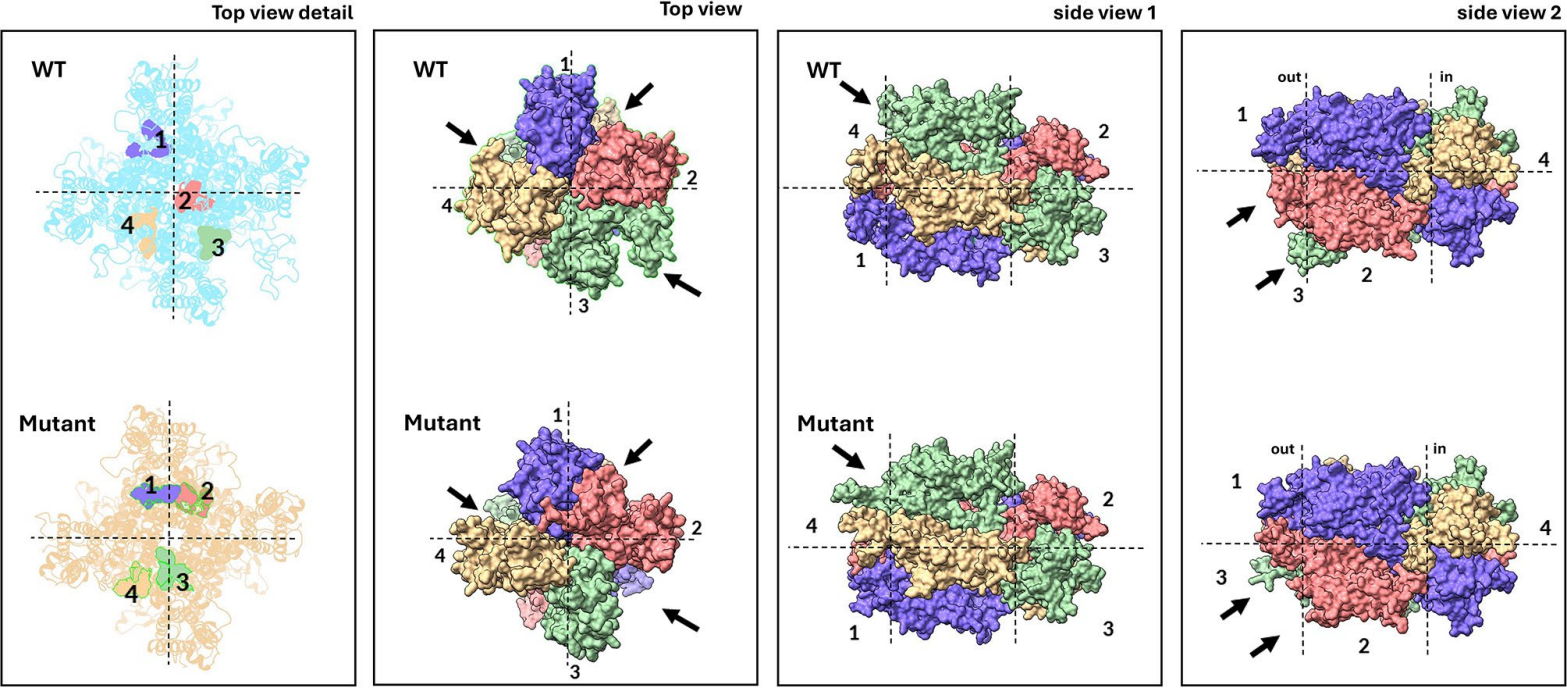
Comparison of 3D structures of DND1 (tetramer) between wild-type (WT) Moneymaker and *dnd1* mutant TV181448-9, indicating the four monomers of the DND1 protein with different colours/numbers (1–4). (**a**) Top view with highlighted 4 amino acids (YGIY) following the F369P370 dipeptide (deleted in the mutant). (**b**) Top view; (**c**) Side view 1; (**d**) Side view 2; in both top and side views some relevant conformational changes are highlighted (black arrows)

To evaluate any conformational change impacting function, we tried to reconstruct the 3D protein structures of both wild-type and mutants. Homology models for both the wild-type and mutated proteins were built and positively validated. The wild-type protein showed a QMEAN4 value of -2.27, and the Ramachandran plot showed that 90.51% of the residues were in favored regions. The QMEAN4 value for the mutated protein was − 2.44, and 90.16% of the residues were in the favoured regions of the Ramachandran plot. The difference between the two models was analyzed in the UCSF Chimera software [50]. A comparison of the 3D structures of both reference and mutant was attempted using the reconstructed 3D proteins in monomer form (Fig. 9) and tetrameric form (Fig. 10). Three observations were made: (1) the mutation (FP369-370del) is adjacent to the selective filter ( [51]; Figs. 8 and 9); (2) the portion of the protein affected by the mutation changes conformation upstream of the selective filter and the pore helix (Fig. 8); (3) this conformational change is sterically bulky (Fig. 9). In the tetramer model, this conformational change is even more noticeable (Fig. 10).

## Discussion

Plants with constitutive defense responses may result from a disabled DND1 function; however, tomato plants with strongly silenced *DND1* expression show pleiotropic effects, are ineffective in fruit production, and have low fitness [36]. In this study, we generated knock-out (truncated proteins) *dnd1* mutants and a *dnd1* mutant with deletion of 3 amino acids (Q114del; FP369-370del) in the tomato cultivar MM susceptible to PM by applying CRISPR/Cas9 technology. We investigated two ways to minimize the negative effects of *dnd1* mutants for breeding: (1) exploiting heterozygous knock-out mutants, to test the potential increased resistance towards *Oidium neolycopersici*, and (2) deepening the study of a unique *dnd1* mutant with deletion of 3 amino acids. The latter was also studied through whole-genome sequencing to exclude the emergence of any unintended off-target effects and to assess its substantial equivalence with wild-type plants.

### Pleiotropic effects of *Sldnd1* mutants: gene-dosage dependence

In previous studies it was shown that CRISPR-KO and RNAi-triggered knock down (RNAi-KD) of *DND1* can result in severe dwarfism, auto-necrosis, and reduced male fertility in different plant species [33, 36, 38]. Recently, three cyclic nucleotide gated channels (CNGC2 – DND1, CNGC4, and CNGC6) null mutants, likely interacting in the formation of a multimeric CNG channel complex, were evaluated [52]; the loss of CNGC6 does not cause dwarfism as the *cngc2* and *cngc4* mutants did. RNAi-KD of *DND1* in tetraploid potato resulted in slight dwarfism [38], suggesting that polyploid plants exhibit better tolerance to decreased expression level of *DND1* than diploid plants probably due to a gene dosage-effect. In this study, T_F2_ progenies showing segregation of CRISPR-induced mutant alleles at the *DND1* locus were developed. This approach was attempted to: (1) overcome the difficulty of selfing the primary mutant transformants; (2) test whether heterozygous *DND1dnd1* plants (containing one copy of the mutant allele) provide adequate PM resistance without showing reduced fitness.

The degree of PM resistance was not in proportion to the copy number of a mutant allele of *SlDND1* in any of the T_F2_ families (derived from E1, E3, E4). Homozygous KO mutants provided protection against PM disease, but neither heterozygous nor wild-type plants did, as highlighted by the analysis of the DI score and fungal biomass. In contrast, *Sldnd1* dosage-dependent dwarfism [53] was demonstrated in segregating families from E1 and E4, but not in the E3-derived family.

### PM resistance in E1 and E4: potential role of phytohormones

Homozygous T_F2_ mutants from E1 and E4, having truncated copies of the DND1 protein because of an early stop codon, lead to a full functional KO of DND1 (Fig. 4). This study shows that PM resistance in tomato can be efficiently achieved by complete KO of *SlDND1*. This is in line with several recent studies that describe resistance to various pathogens through RNAi silencing and CRISPR editing of *DND1* in different crops [37, 54].

The *dnd1* mutants of *Arabidopsis* conferring a broad-spectrum resistance failed to produce HR [33]. This was accomplished by sustaining high levels of defence-associated phytohormones, such as the constitutive expression of pathogenesis-related (PR) genes [33]. For monitoring the activity of defense-related signalling pathways, expression profiles of phytohormone marker genes have been investigated in a number of studies concerning different plant-microbe interactions. In *Arabidopsis dnd1* mutants only the SA-mediated signalling pathway illustrated by elevated expression of the SA-dependent *PR-1* gene contributes to increased resistance to *Pseudomonas syringae* pv. *tomato* DC3000 [33] and *Botrytis cinerea* [55]. The increased resistance to late blight caused by *P. infestans* observed in RNAi-*DND1* silenced potato plants relied on an early induction of both the SA- and ET-mediated signalling pathways [37]. To understand the mechanisms underlying PM resistance in tomato, it will therefore be worthwhile in the future to investigate expression profiles of SA, ET, and JA pathway marker genes in *dnd1* KO mutants.

### Fitness advantages of *Sldnd1* E3 allele: homology modelling and 3D structure

Homozygous mutants from E3 line represent the first example of induced mutation in *DND1* generating tolerance to PM with less negative pleiotropic effects. To assess the structural basis for this unique trait, homology modelling of DND1 in the E3 event as well as in the other events (E1 and E4) and in the wild-type MM sequence was carried out and discussed.

The protein CNGC2, encoded by *DND1*, senses fluctuations in the levels of intracellular cyclic nucleotides (cNMP) and controls a variety of cellular processes, notably the influx of Ca2 + into plant cells [51]. Recent evidence strongly supports the role of cyclic nucleotide-gated channels as primary effectors of cNMPs in plant cells [51]. These channels serve as crucial cellular switches, transducing variations in the intracellular levels of cyclic nucleotides into alterations in membrane potential and ion concentrations [56].

The E3 allele in TV181448-9 mutant can produce auto-necrotic spots on the leaves, so it might maintain the capability to produce HR. The DND1 protein structure suggests an involvement of the region related to the cation sensing (selectively filter + pore domain), since the mutation in E3 impacts, from the structural point of view, the part of the protein related to the pore-loop cation channel (Figs. 9 and 10) exhibiting relevant conformational changes (Fig. 10**).** In cyclic nucleotide-gated channels, the “selective filter” is a critical region within the pore domain that dictates ion selectivity [51]. This filter enables the channel to selectively permit the passage of specific ions (e.g., Ca2+, K+, Na+) based on their size and charge, while excluding others. Conformational changes in ion channels often affect gating mechanisms that control their opening and closing. For example, in voltage-gated potassium channels, changes in voltage sensor domains open the channel pore [57]. Similarly, conformational changes in the CNG channel of DND1 may indicate how mutations alter channel opening and ion flow. In particular, the two-amino-acid FP369-370 deletion in the DND1 protein in the TV181448-9 mutant, may cause a conformational change in the protein’s pore-loop cation channel, which affects the recognition of Ca^2+^ ions, leading to dysregulation of Ca^2+^ signalling.

The mutation FP369-370del is located adjacent to the selective filter of the CNG channel. The selective filter is crucial for the channel’s function, determining the selectivity and conductance properties of the channel. Mutations in or near this region can significantly alter how ions pass through the channel, potentially affecting the channel’s ion selectivity and gating properties. Steric bulk can hinder the movement of protein parts essential for channel function. For instance, in the MscL channel of *Escherichia coli*, bulky side chains were found to affect channel gating by blocking or altering necessary movements within the channel structure [58]. This proximity suggests that the FP369-370del mutation could disrupt normal ion flow, leading to altered cellular functions or signaling pathways. Moreover, in a tetrameric model, the mutation-induced conformational change is more pronounced, suggesting magnified effects in the quaternary structure. Cooperative behavior in tetrameric channels, like CNG and Shaker potassium channels, means changes in one subunit can influence others, altering overall channel dynamics and function [59].

Thus, it would seem appropriate to investigate the role that “selectively filter” plays in the DND1 protein of the TV181448-9 mutant and the role that the conformational change, the steric bulkiness of this change and/or the amplification effect of the change in the tetrameric model might play in cation (Ca^2+^) recognition. It would be interesting to conduct affinity experiments on different cations using the mutant gene and wild-type.

### Breeding values of homozygous E3 line

Although the CRISPR/Cas9 approach can result in random mutations at target loci that are functionally equivalent to natural mutations, it is not always easy to predict this equivalence. It has been suggested that the variations observed in edited lines are mostly induced by somaclonal variation during in vitro culture, inheritance from maternal plants, and pre-existing variation across the germline [60]. Whole genome sequencing (WGS) can be used to analyze the substantial equivalence of edited lines with their wild-type counterparts. WGS provides comprehensive information about genomic variations, such as indels, SNPs, other structural differences, and the presence/position of *Cas9* residual copies. Several studies have employed WGS analysis of WT and CRISPR/Cas9-edited lines to investigate the specificity of genome editing [61]. These studies observed that off-target mutations occur at a much lower level than background mutations due to pre-existing/inherent genetic or/and somaclonal variations [24, 60, 62–64].

In agreement with these observations, targeted deep sequencing of *sldnd1* (E3, TV181448-9) mutant plant at 28 putative off-target loci confirmed the absence of off-target mutations (Table S5 and S6). The average number of SNPs and variation rate was comparable between unedited and edited plants (6.34 × 10^− 5^ for edited plants vs. 5.59 × 10^− 5^ for unedited plants, respectively, Table S7) and coherent with what was previously observed in tomato (Li et al. 2022). In addition, our *de novo* genome assembly using the WGS data indicates that the *Cas9* gene could readily be eliminated by segregation in T_F2_. Such elimination from *dnd1* plants classifies them as NGT 1 events (New Genomic Technologies, category 1; [65]) and prevents the induction of mutations at untargeted loci. Homozygous mutants from the E3 line promise to be useful in future breeding projects. Indeed, it will be crucial to evaluate the impact of these genomic variations on agronomically important traits, such as tomato fruit production; for these reasons, comprehensive field trials and phenotypic assessments will be necessary to assess the overall productivity.

## Conclusions

Our results demonstrate, for the first time, a reduced susceptibility to *On* in tomato KO *dnd1* mutants obtained through CRISPR/Cas9 gene editing and describe a special *dnd1* mutant allele, with reduced fitness costs. This plant lost any T-DNA insertion (e.g. eliminated *Cas9*) and showed the presence of a causal mutation (amino acids deletion) in the *DND1* locus, which is virtually indistinguishable from one that might occur in nature. The present findings underscore the importance of precision genetic engineering, as even small changes can have significant impacts on a plant’s overall phenotype. These results align with the broader philosophy of “less is more”, which emphasizes the importance of simplicity and essentiality in design and construction, a principle that can be applied not only to architecture but also to genetic engineering.

### Electronic supplementary material

Below is the link to the electronic supplementary material.

Supplementary Material 1

## Data Availability

The sequence data were deposited in the NCBI Short Read Archive under specific submission identifiers (PRJNA1090062).

## Declarations

### Dual publication

The results/data/figures in this manuscript have not been published elsewhere, nor are they under consideration (from you or one of your Contributing Authors) by another publisher.

### Third party material

All of the material is owned by the authors and/or no permissions are required.
